# An Ontology of Quality Initiatives and a Model for Decentralized, Collaborative Quality Management on the (Semantic) World Wide Web

**DOI:** 10.2196/jmir.3.4.e34

**Published:** 2001-12-31

**Authors:** Gunther Eysenbach

**Keywords:** Semantic Web, Resource Description Framework, World Health Organization, Internet/standards, Ethics, Professional, Social Control, Formal, Health Care Quality, Quality Assurance, Health Care/standards, Commerce/standards, Information Management/standards, Medical Informatics/standards, Quality control

## Abstract

This editorial provides a model of how quality initiatives concerned with health information on the World Wide Web may in the future interact with each other. This vision fits into the evolving "Semantic Web" architecture - ie, the prospective that the World Wide Web may evolve from a mess of unstructured, human-readable information sources into a global knowledge base with an additional layer providing richer and more meaningful relationships between resources. One first prerequisite for forming such a "Semantic Web" or "web of trust" among the players active in quality management of health information is that these initiatives make statements about themselves and about each other in a machine-processable language. I present a concrete model on how this collaboration could look, and provide some recommendations on what the role of the World Health Organization (WHO) and other policy makers in this framework could be.

"An ontology is a specification of a conceptualization,

*i.e., a formal description of the concepts and their relations for a universe of discourse."* [1]

In this issue of the Journal of Medical Internet Research, Risk and Dzenowagis present a review of quality initiatives of health information on the Web [2]. This review will be a useful starting point for anybody interested in this field, with the limitation that it is not a systematic review meant to cover all relevant initiatives worldwide (see Textbox 1 for some additional initiatives not mentioned in the report)

The review raises a question about how the different initiatives relate to each other and how they could play out their potential for synergy to benefit consumers and users. The following article shall provide a framework and an abstract model of how these initiatives may in the near future interact with each other. While there have been many calls for collaboration between the existing initiatives, I will present a concrete schema on how this collaboration could look and also what the role of the World Health Organization (WHO) and other policy makers in this framework could be.

**Additional initiatives not mentioned in the review**

The report of Risk and Dzenowagis [2] focuses on 13 selected initiatives that are most visible in the Western world (eg, through publications and participation in international meetings), namely:

- eHealth Code of Ethics
- Health Internet Ethics (Hi-Ethics)
- URAC Health Web Site Accreditation Program
- MedPICS Certification and Rating of Trustworthy and Assessed Health Information on the Net (MedCERTAIN)
- TNO Quality Medical Information and Communication (QMIC)
- Health on the Net Foundation Code (HON Code)
- EC (European Community) Quality Criteria for Health-related Websites
- Organizing Medical Networked Information (OMNI)
- DISCERN
- American Medical Association (AMA): Guidelines for Medical and Health Information Sites on the Internet: Principles Governing AMA Web Sites
- British Healthcare Internet Association (BHIA): Quality Standards for Medical Publishing on the Web
- The Health Summit Working Group-Criteria for Assessing the Quality of Health Information on the Internet: IQ Tool (HSWG IQ Tool)
- The International Federation of Pharmaceutical Manufacturers Associations (IFPMA) Code of Marketing

Some additional initiatives are worth mentioning, for example:

- Third-party certification programmes:
  - the Japanese "JIMA mark" [3],
  - the Verified Internet Pharmacy Practice Sites (VIPPS) certification mark of the US National Association of Boards of Pharmacy [4]
  - the Web Médica Acreditada initiative of the Medical College Barcelona
- Third-party annotators and gateways, such as HealthInSite or Healthfinder
- Groups and organizations active in promoting quality standards or codes of conduct, eg. national health-information-provider associations such as *Aktionsforum Gesundheitsinformationssytem*(AFGIS) [5] or AMIDI in Germany [6], the American Health Information Management Association (AHIMA) [7] or the European Federation of Pharmaceutical Industries and Associations (EFPIA) [8].

The Journal of Medical Internet Research encourages these and other initiatives and organizations not yet listed here to submit letters or articles to present themselves.

## "Why bother at all? We don't care about the quality in other media either"

One frequent question asked is why we should look at quality issues on the Web at all: there is also misinformation in other media where we seem to do little to ascertain the quality. Risk and Dzenowagis also quote the argument that "Traditional media did not require quality standards; therefore neither should the new media." However, I can see at least 4 reasons why this is not a convincing argument:

- First, the fact that we have not done something in the past is hardly a sufficient argument for not doing something in the future. The quality of patient education and consumer health information has been a neglected field over the past decades, and this should not be an argument for continuing this negligence.
- Second, it is simply not true that nothing is being done in traditional media, as there are quality standards and codes of conduct for traditional media as well; there are also evaluators guiding us to high-quality information such as television guides, and book reviews; there are also organizations that for example certify printed patient-information leaflets.
- Thirdly, there are several characteristics of the Internet which make information and communication over this medium "special" and attention to quality issues necessary, in particular:
  - (1) lack of quality control (editorial boards) at the stage of production is more prevalent than in traditional media *;*
  - (2) the extremely cheap publishing process makes it easy to publish without the need to make revenue, thus without the need to stick to highest publishing standards;
  - (3) dubious and alternative medicine products are now primarily offered on the Internet;
  - (4) a " *context deficit*", leading to the situation that information does not necessarily have to be false to harm [9];
  - (5) enormous reach, with the potential to affect the health of large populations;
  - (6) interactivity, leading to higher involvement of the users and perhaps a greater impact on individuals;
  - (7) users retrieve information "just-in-time" and are more likely to apply it immediately. Unlike information in other media, which often is encountered by consumers only by chance, users on the Web mostly retrieve information "on demand"-when they need a piece of information, they type the respective search terms into a search engine, and are likely to act immediately upon the information they searched for.
- A fourth reason - and perhaps the most important - is that the Internet is not a static medium such as a patient leaflet, a newspaper, or a book, where once a person has obtained misinformation there is little health professionals can do to complement or rectify this information. On a decentralized, electronic medium, intelligent systems can automatically give additional information about the information from other sources to the consumer, or help in guiding consumers to the best-available evidence. In the future, people will use intelligent browser plug-ins for "knowledge based" Web-browsing, as well as intelligent software agents that retrieve information using metadata (data about data) harvested from the "semantic" web. It is this vision I am going to dwell on in the following.

## The need for intelligent "next generation" tools

The common, overarching aim of any quality initiative is the desire to "help people, patients and professionals to identify health information useful to them" [10]. As the Risk and Dzenowagis review shows, there is a lack of reliable and valid tools that can be used by consumers or professionals to locate trustworthy health information. Neither questionnaire instruments such as DISCERN nor "kitemarks" (in the form of simple seals or logos) provide appropriate and sufficient ways for consumers to assess the trustworthiness of information (letting alone the problem that some consumers may ignore or not have the skills to look at and interpret the correct quality markers). Kitemarks and questionnaire instruments very much come from traditions outside of the Web and do not harness any of the advantages of the Web as a decentralized information system. There is a need for "next generation" tools, intelligent knowledge-based tools, allowing consumers to positively identify reliable health information suitable for their needs, such as intelligent agents or client-side advisory systems for people. These intelligent tools will be able to aggregate and process statements (descriptions, annotations and ratings) made by a variety of actors and integrate them with the individual preferences of the user, thereby harnessing the power of the Web as a decentralized medium. These statements (descriptions, annotations, and ratings) are essentially "data about data", or "metadata" (for an excellent introduction into metadata see [11]), and they are the prerequisite for forming the semantic web, which "will bring structure to the meaningful content of Web pages, creating an environment where software agents roaming from page to page can readily carry out sophisticated tasks for users." [12]

## The actors

Many individuals and organizations ("actors") from the health care field have become interested in the topic of quality of health information on the Internet. This interest usually arises out of one or more of the following motivations or perspectives:

- An individual or organization is (or wants to become) a "health information provider" ("first party"). Health information providers are usually interested in providing health information or services on the Web according to the highest-possible quality standards, and want to know what quality criteria they should adhere to, eg, what information they should disclose, and whether or not they act in line with generally-accepted quality guidelines or codes of conduct. These individuals or organizations may also be interested in using quality as a marketing argument, eg, by displaying to the user that they adhere to these standards, especially if the health information provider hasn't yet established a brand name which the user associates with quality. Ideally, this quality mark is not self-awarded but indicates that an independent party (a "third party," see below) has confirmed adherence to predefined standards.
- An end user ("second party") wants to know whether or not to trust information, and wants to know what quality criteria or quality marker he or she should look at.
- An independent individual or organization ("third party") feels special responsibility or has special expertise and knowledge to endorse, evaluate, validate, certify, recommend, approve, peer-review, comment on, or annotate information or services provided by health information providers (or other actors). These third parties could be, for example, gateways, libraries, portal sites, or certifying institutions.
- An organization or association ( *group*) of health information providers ("fourth party") wants to set up a code of conduct or guideline, eg. because it has responsibilities for its members and wants to set-up guidelines or codes of conduct for these members to comply to.

In practice, each of these actors can have one or more of these roles simultaneously, for example, an evaluating third party can be identical to the actor that sets up codes of conduct (fourth party).

## The framework

**Figure 1:**
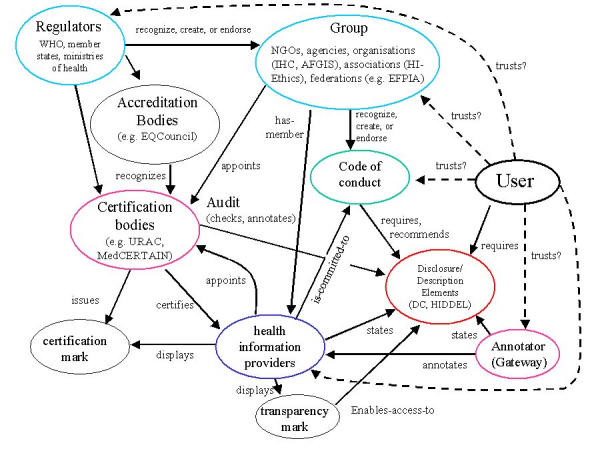
A (simplified) model of decentralized quality management ("Collaboration Schema") or "web of trust". Actors in this collaboration use metadata (eg. expressed in XML/RDF) to describe their relations with other actors and to make statements about themselves or other actors using elements from standardized vocabularies (DC=Dublin Core, HIDDEL=Health Information Disclosure, Description and Evaluation Language). Users can set their own information preferences and requirements using the same vocabularies, and/or can tell their software that they trust certain actors a-priori (dashed lines). Intelligent browsing tools or agents may then assist users to locate trustworthy information

I now describe some of the roles these actors can have in a decentralized "health information quality management framework." In Figure 1, this framework is depicted by illustrating the actors or other concepts as nodes (in the description below the nodes are in italics) and the relationships between the actors as arcs (underlined).

In this framework, there will be the following concepts and relationships:

- *health information providers*(blue) which are for example "committed-to" a set of *codes of conduct*, ie, to a standard, or guideline (green). For instance, a *health information provider* could be committed-to the eHealth Code of Ethics [13]. At the same time, health information providers adhere to the disclosure and transparency recommendations in these codes of conduct by making certain *statements* about themselves. These statements (for instance, a disclosure statement about who the sponsor of the site is) can (and should) not only be expressed in "narrative" form, but in a standardized, machine-readable form.
- *groups*(light blue), ie, organizations or associations, which can "have-members." Groups are, for instance, associations, federations, or other organizations (eg, Hi-Ethics, AFGIS, IFMPA/EFPIA); their members usually are *health information providers*. The group as a whole may create or endorse *codes of conduct*(green), implying that their members are supposed to stick to this standard.
- these *codes of conduct*(created or endorsed by a group) in turn contain items that may, for example, prescribe the existence of certain d *isclosure and description elements* on a Web site (such as the disclosure on who the sponsors are), or meeting certain other requirements such as a maximum response time for email requests, the recognition of another body (eg. licensed cyberpharmacies) etc.
- *groups* or individual *health information providers* optionally may appoint external *certification organizations* that may give additional assurance to users, to the health information providers themselves, or to the groups representing health information providers, that their members actually stick to their self-prescribed standards. An audit by a third party may be a necessary "enforcement" mechanism to prevent users from getting a false sense of security when relying just on self-labelling or self-commitment. Some *members* claiming to be committed to *codes of conduct* may fall short of the principles prescribed therein and may damage both the reputation of the code and of the group promoting it. In more tightly-regulated areas, such as in the pharmaceutical industry, associations will have an intense interest in avoiding situations where individual members violate-willingly or unwillingly-their self-regulatory codes, as this may trigger regulators to step in and to replace self-regulation with legislation. The certification process can be seen as the solicitation of third-party statements which will complement, validate, or comment the statements made by the health information provider, empowering the consumer to compare what the health information provider claims with what an independent party says. Thus, a *group* or *health information provider* could appoint or hire a *certification body* to conduct an audit, which would involve the certification body in making statements about statements (eg, to check or annotate disclosure statements made by the health information provider), or simply confirming (certifying) that the health information provider complies with the prescribed guideline as a whole (if the health information provider does not pass the audit it is up to the *group* to decide on possible sanctions such as withdrawal of membership if the health information provider falls short of the *code of conduct*). Such an audit could be done by humans, or by software, or by a combination of both (in the Semantic Web scenario it would be easy to make certain checks automatically, eg, to check whether certain disclosure statements prescribed in the guideline are present, but it may be advisable to check the content by humans as well). It is important that the certifier is explicit about which aspects of the site have been checked, by whom, and when. Traditional "kitemark" approaches, simply relying on a logo or seal, often fall short of reaching this explicitness, which can however be reached again by making RDF statements (RDF=Resource Description Framework, an infrastructure for organizing and managing metadata [14]) about the RDF statements made by the health information provider.
- In addition, we may see the emergence of accreditation bodies, which "accredit" (ie, endorse or recognize) certifiers. (Note that we discriminate the terms certification and accreditation here - what a certifier like URAC presently calls "Web site accreditation" is, according to this terminology, actually certification). For example, a MedCERTAIN steering group may decide to "accredit" (recognize, endorse, support) other certifying organizations that act according to the MedCERTAIN model, ie, demand machine-readable level-1 descriptions from their members (disclosure and self-description labels) and perform level-2 and level-3 descriptions (provide computer-readable evaluative metadata from third parties).

## Statements made by actors

One key issue for interlinking these players on the Semantic Web is that they speak a common language. With this language, these actors may say certain things about themselves and each other, like:

- Health information provider A (first party): "I am a member of an organization called D. I am committed to answer all my e-mails within 3 days. I am funded by public money. My target audience is consumers, my information is provided in English, and my main internal quality-assurance mechanism is described on page URL."
- User B (second party): "I trust organization E, but I don't know whether or not I can trust health information provider A. I prefer to have health information providers that are located in Germany and I prefer health information providers that answer my e-mail questions within 3 days."
- Certifier C (third party): "I can certify that health information provider A complies with the standards set up by group D."
- Group D (fourth party): "I am an organization with the name D, I am sponsored by S, and we have adopted guideline Z. We have appointed an external certification agency C to audit our members and to make sure that they actually stick to these codes of conduct."
- Organization E (Accreditor): "I am recognizing certification body C."

These actors form a complex network in making statements about each other or about themselves. Transparency is one of the ethical tenets demanded by all ethical codes, but how transparent is this complex network in reality to the user, if the actors use only human-readable (not machine-processable) information? For a human user, it may be almost impossible to figure out the various relations between these players and to infer from the statements, eg, to conclude whether or not the user can trust a given health information provider (leaving aside the difficulty of obtaining these statements in a timely manner). In fact, some "intelligence" and "reasoning" (analyzing the relationships and their implications) is necessary. The multitude and complexity of the relations between the initiatives and the data they produce will soon be too complex to be interpreted and digested by consumers without intelligent systems helping them to infer from what the various initiatives say. The consumer will need intelligent systems (browser plug-ins or intelligent agents), which the user can feed with some information on the his/her information-quality needs, for downstream filtering, eg, advising whether or not to trust a given site.

## Towards a Health Information Disclosure, Description and Evaluation Language

It is the vision of the protagonists of the Semantic Web to form a consistent logical web of data on the World Wide Web. A prerequisite for the Semantic Web is the development of languages for expressing information in a machine-processable form. In line with this vision, one aspect of the MedCERTAIN project [15] aims to harness the power of networked information to achieve decentralized quality management and to weave a machine-processable web of trust. The first step was therefore the definition of a language to express self-descriptions and third-party annotations for health Web sites, formerly called medPICS [16], now called HIDDEL (Health Information Disclosure, Description and Evaluation Language) [17]. This language can be expressed in XML/RDF and can be used to "label" sites in a standardized format; similar to food labels [18,22]. Web sites could carry a machine-readable file (eg, hiddel.xml) which can be parsed and processed by software. For example, the statements by group D made above could be expressed using HIDDEL vocabulary elements and XML/RDF syntax as depicted in Textbox 2 (XML means eXtensible Markup Language).

Software designed to assist users in locating trustworthy information also needs some additional "knowledge", such as how the actors relate to each other and what these relations imply; for example, the fact that if a *health information provider* is a member of a *group*, this implies that the provider is committed to (and supposed to) stick to the guideline adopted by the group. Any such framework (or ontology) can be expressed as a "schema" in a Semantic Web language such as RDF or DAML/OIL (DARPA Agent Markup Language), and indeed Figure 1 is a simplified version of an RDF schema modelled in the MedCERTAIN project. Such a schema can serve as a template for each of the actors to make statements about themselves and other actors, and more importantly, it would allow "knowledge" to be given to intelligent client-side software or intelligent agents to query this semantic network and to make inferences, eg, about the trustworthiness of given actors based on what others say about them and what they say about themselves.

The Health Information Disclosure, Description and Evaluation Language therefore has 3 components:

- A HIDDEL core vocabulary: hierarchical metadata elements and subelements, providing the predicate in an RDF statement to describe properties of resources, eg, to indicate a sponsor. This metadata vocabulary is different from generic vocabularies such as the Dublin Core, as it uses atomic terms and concepts from ethical codes such as the eHealth Code of Ethics and includes concepts normally only used by third parties to describe or evaluate health Web sites. It also enables, for example, health information providers to make disclosure statements in a machine-readable form [17].
- A "collaboration schema" modelling a collaborative framework, giving names to the actors and defining their relationships (as, in a simplified form, depicted in Figure 1).
- An "annotations schema," providing a mechanism for making statements about statements [19].

The development of HIDDEL is an ongoing process requiring the continuous input of all organizations active in the field. We have previously attempted to draw together these players for an initial workshop in Heidelberg to agree on some building blocks for a core vocabulary and ontology that can be used on a Semantic Web [20], and a second workshop will be hosted in 2002. We formerly called this (informal and loosely organized) community "Collaboration for Critical Appraisal of Health Information on the Internet" [9] and now refer to it as the "Heidelberg Collaboration" [10,18]. There is no need for political wrangling and wrestling among organizations about under whose umbrella a collaboration should take place and who should take the lead in the hierarchy - a hierarchy doesn't exist on the Web. Or as Tim Berners-Lee put it: "That's the beauty of the Web: It's a web, not a hierarchy" [21].

**Example machine-readable site-label in XML/RDF and HIDDEL (also using a Dublin Core element), as it could be provided on a website of an association of health information providers . The label says the following: "I am an organization with the name D, I am sponsored by S, and we have adopted guideline Z. We have appointed an external certification agency C to audit our members A, B and C.". Similar labels can be used by other health information providers to make machine-readable disclosure or description statements. [Note: this is for illustration purposes only - the HIDDEL specification is still under development and elements may change].**

<?xml version="1.0"?>

<RDF xmlns = "http://www.w3.org/1999/02/22-rdf-syntax-ns#"

xmlns:rdf="http://www.w3.org/1999/02/22-rdf-syntax-ns#"

xmlns:DC = "http://purl.org/dc/elements/1.1/"

xmlns:HIDDEL = "http://www.medcertain.org/metadata/2001/12/HIDDEL#">

<Description rdf:about=" **D** ">

<DC:title> **Group D** </DC:title>

<HIDDEL:sitespecific>

<HIDDEL:disclosure>

<HIDDEL:funding> **Sponsor S** </HIDDEL:funding>

</HIDDEL:disclosure>

</HIDDEL:sitespecific>

<HIDDEL:endorsed-guideline> **Z** </HIDDEL:endorsed-guideline>

<HIDDEL:appoints-certifier> **C** </HIDDEL:appoints-certifier>

<HIDDEL:has-member>

<Bag>

<li> **A** </li>

<li> **B** </li>

<li> **C** </li>

(...)

</Bag>

</HIDDEL:has-member>

</Description>

</RDF>

## The role of the World Health Organization and policy makers

Only a few weeks after the first Heidelberg workshop, WHO brought forward the "dot-health" proposal [22]. A quote from a WHO representative reveals the level of confusion on Internet standards: "A top-level domain, as a recognized Internet label, is more valuable than a trustmark because of its enforceability. . .it can be suspended or canceled if the domain-name holder is in violation of the established standards. The .health top-level domain has the potential to become a reference model for how international organizations and other, non-technology focused groups can support and promote transparent, high-quality information on the Internet in their respective fields." [23]

This statement not only shows a certain degree of naivety on the difficulties of withdrawing a domain name (which would have disastrous effects on a health information provider and would inevitably lead to legal battles) as opposed to a trustmark, it also indicates that WHO was very much thinking in terms of hierarchies and failed to recognize fundamental design principles of the Web as a decentralized, non-hierarchical medium, and that top-level domains never were thought of as "quality labels." Instead, the W3C (World Wide Web Consortium) recommendation for endorsement data ("labels") was the PICS standard, which is now being replaced by XML/RDF [14].

So what is the role of WHO and health policy makers in this framework? I would add the following recommendations to those already made in the Risk and Dzenowagis paper:

### Recommendation 1: Take on the role as an actor in the Semantic Web

First, WHO can take on the roles of any of the actors described above, being part of a *web* rather than attempting to form the top of a hierarchy. As one player in the Semantic Web, WHO could, for example, endorse or appoint any other actor - and make these endorsements explicit on the Semantic Web using RDF metadata. For example, WHO could use metadata on its site to link to trusted government sites of member states or to Web sites of NGOs (nongovernmental organizations) which have official relations with WHO (in this scenario, WHO would act as the leader of a *group* according to the schema defined above). As such, it may for example also create (or endorse) a guideline for Web sites of the organizations that have official relations with WHO, and could "enforce" this code of conduct by appointing an external certification organization. One of the certification criteria could be that these organizations use metadata to identify the sites they trust, and demand the use of metadata on the sites they trust, and so on. Consumers could then parse which organizations are trusted by the WHO, whom these organizations trust, and so on - thereby forming a web of trust.

### Recommendation 2: Make health information on the Web a policy priority

Secondly, acknowledging that the quality of health information is a critically-important public health issue, as it could potentially affect health outcomes for millions [24], health information on the Web should be made a WHO program priority and - recognizing that research is urgently needed in this field - WHO should also consider designating one or several "Collaborating Centers for Consumer Health Informatics." In other fields, WHO has acknowledged that research in policy priority areas is best advanced by assisting, coordinating, and making use of the activities of existing institutions, and has appointed collaborating centers, eg, for the purpose of standardization of terminology, nomenclature, technologies, methods and procedures.

### Recommendation 3: Promote best practices, including the use of metadata

Thirdly, perhaps the biggest role for WHO (and policy makers in member states) is the promotion of the appropriate standards (rather than promoting the wrong hierarchical models - see comments in this section about "dot-health"), and of best e-health practices. This includes that the provision of metadata (for disclosure and description) should be promoted as being one important quality criterion for Web sites *per se*, and that WHO should act as a role model in providing and using metadata itself (see recommendation 1).

Promotion and backing of this approach from the policy side is needed, as otherwise uptake of Semantic Web technologies in the health field could be delayed by a typical chicken and egg problem: If health information providers (and the other actors such as third-party gateways) do not start using RDF metadata, there will be no vendors developing semantic-web/web-of-trust applications. If there are no applications, health information providers will have no incentives to use RDF metadata. The medical community is currently wasting too much time and effort with debating anarchical quality-control mechanisms - such as seals of approval - and with politicized discussions on who should be in charge of quality control, without recognizing that the Web itself provides the answer. The first Heidelberg workshop [20] provided the best example: A workshop designed to debate a metadata vocabulary was quickly overturned by a general debate about who should be in charge and whether we should provide evaluative data at all.

Publicly-funded projects such as MedCERTAIN (MedPICS Certification and Rating of Trustful and Assessed Health Information on the Net, 2000-2001) and MedCIRCLE (Collaboration for Internet Rating, Certification, Labeling and Evaluation of Health Information, 2002-2003) aim to create awareness and a critical mass of metadata, so that industry jumps in and develops intelligent Web browsers and agents able to aggregate and interpret this data. Still, MedCERTAIN is often misunderstood as a third-party certification service or trustmark project, on par with, eg, URAC. However, although this is one aspect of the project, the main goal of the project is to demonstrate the overall framework depicted in Figure 1 and to demonstrate the use of metadata. In the follow-up project MedCIRCLE, 3 European gateways will implement HIDDEL on a broader scale, will demonstrate the synergy created through collaboration on the Semantic Web, and will invite and support other organizations to become part of the "Heidelberg Collaboration" by implementing the HIDDEL vocabulary.

### Conclusion and outlook

The Semantic Web will greatly magnify the challenges, but also the opportunities, created by the human-readable World Wide Web. On the Semantic Web, people will use intelligent agents to find the cheapest airfares or the best used car in town, but inevitably they will also ask intelligent agents about the best physician or best treatments available. It is easy to imagine what will happen without quality assessment and quality-related metadata: "intelligent" agents will not deliver the best medical answers, but may provide answers given on quackery sites. Without quality related metadata, the impact of the Semantic Web on consumers could be detrimental. On the opportunity side, the Semantic Web will give even greater power to the consumer to determine the trustworthiness of a given health information provider or service than the Web in its current form, if quality-related metadata are used. The Semantic Web also opens up new ways for educating consumers and reaching less technology-savvy and health-literate consumers, because part of the intelligence and knowledge required to critically appraise and understand health information (and to put it into context with one's personal health data) could be built into search tools and client-side software.

While the biggest advantage of the Semantic Web is often discussed under the aspect of increasing the findability of information ("resource discovery"), and while this may remain to be an important aspect for health information on the Web, the perhaps bigger opportunity for e-health lies in the prospect of weaving a web of trust. The e-health community has the unique opportunity to lead this development, where much research and standardization work needs to be done.

With this perspective in mind, the time is ripe for the health information quality initiatives to start looking beyond their own horizon and to become active as a player in the Semantic Web.

## Abbreviations

DAML/OIL: DARPA Agent Markup Language / Ontology Inference Layer
HIDDEL: Health Information Disclosure, Description and Evaluation Language
MedCERTAIN: MedPICS Certification and Rating of Trustful and Assessed Health Information on the Net
MedCIRCLE: Collaboration for Internet Rating, Certification, Labeling and Evaluation of Health Information
MedPICS: Platform for Medical Content Selection
NGO: Nongovernmental Organization
PICS: Platform for Content Selection
RDF: Resource Description Framework
WHO: World Health Organization
XML: eXtensible Markup Language
